# StressFoot: Uncovering the Potential of the Foot for Acute Stress Sensing in Sitting Posture

**DOI:** 10.3390/s20102882

**Published:** 2020-05-19

**Authors:** Don Samitha Elvitigala, Denys J. C. Matthies, Suranga Nanayakkara

**Affiliations:** 1 Augmented Human Lab, Auckland Bioengineering Institute, The University of Auckland, Auckland 1010, New Zealand; denys.matthies@th-luebeck.de (D.J.C.M.); suranga@ahlab.org (S.N.); 2 Department of Electrical Engineering and Computer Science, Technical University of Applied Sciences Lübeck, 23562 Lübeck, Germany

**Keywords:** stress sensing, smart insoles, smart shoes, unobtrusive sensing, stress, center of pressure

## Abstract

Stress is a naturally occurring psychological response and identifiable by several body signs. We propose a novel way to discriminate acute stress and relaxation, using movement and posture characteristics of the foot. Based on data collected from 23 participants performing tasks that induced stress and relaxation, we developed several machine learning models to construct the validity of our method. We tested our models in another study with 11 additional participants. The results demonstrated replicability with an overall accuracy of 87%. To also demonstrate external validity, we conducted a field study with 10 participants, performing their usual everyday office tasks over a working day. The results showed substantial robustness. We describe ten significant features in detail to enable an easy replication of our models.

## 1. Introduction

Stress has a direct impact on our well-being [1]. Although stress is often perceived negatively, it can have positive aspects. For instance, acute stress aims to mentally and physically prepare our body to accomplish a demanding task. In contrast, episodic occurring acute stress can cause a variety of negative symptoms, such as sleep disorders, headache, stomach pain and exhaustion. A constant elevated stress level may even result in chronic stress [2], which can lead to severe health conditions, such as depression, anxiety, hypertension and cardiovascular diseases [3]. Therefore, it is imperative to identify stressful situations to prevent resulting illnesses.

Previous studies have demonstrated the capability of identifying stress based on physiological parameters [4], such as electrodermal activity (EDA) [5], heart rate variability (HRV), brainwaves via electroencephalography (EEG) [6,7,8], muscle tension [9,10], facial expressions [11] and body language [12,13], as well as self-reporting [14,15]. Although these methods provide reliable stress indicators, there are drawbacks. For instance, sensing physiological parameters are sensitive to movement artifacts. The sensing device also needs to be instrumented tightly on the user’s body, resulting in low comfort. An alternative method is contact free sensing, such as using cameras [16,17,18]. However, these systems suffer from varying lighting conditions, require line of sight, and typically create privacy concerns.

Manual approaches, such as having an experimenter interpret facial expressions and body language or relying on self-reporting, are prone to issues such as scalability and subjective bias. Several other studies explore stress detection based on smartphone usage [19,20,21] by correlating screen-time with daytime, and utilising the phone’s sensor data. These studies show the capability of detecting stress over long periods of time. Identifying and predicting acute stress in the short-term may also be possible, although still problematic [21].

Smart shoes, in particular insoles, have been used in a variety of scenarios, such as to analyse gait [22], identify postures [23], calculate walking speeds [24], determine the ground surface [25] and recognising foot tapping gestures for an interaction control [26]. These smart insoles provide an unobtrusive way of collecting data. However, to our knowledge, insole-based tracking has not yet been proposed to identify stress.

Motivated by the fact that feet and legs may carry essential information about a person’s stress level [12,27] and the widespread availability of shoes, we present StressFoot. Our prototype encapsulates a smart shoe system that incorporates a pressure-sensitive insole based on force-sensitive resistor (FSR) technology and an inertial measurement unit (IMU). We drive a machine learning approach to reliably detect acute stress situations in sitting postures, such as sedentary office work. To scientifically validate this, we followed the standard research design process [28] by Constructing Validity—Study 1 developing a machine learning model based on the data of 23 participants, evidencing Empirical Replicability—Study 2 with 11 participants showing a distinguishability between stressed and relaxed conditions and finally, testing for External Validity—Study 3 showing the generalisability in terms of robustness of our model for office workers during a typical 8 h work day with 10 participants. In summary, we contribute:

- a novel way to discriminate acute stress and relaxation by four distinct foot movements and posture characteristics,
- ten mathematical features to train machine learning models,
- and design implications for future applications in ubiquitous computing.

### 1.1. Background

#### 1.1.1. Physiological Responses

The most common approach in stress sensing is interpreting physiological responses, such as EDA [29], heart rate [30] (HR), HRV [31], pupil dilation [32] (PD), skin temperature [33,34] (ST) or EEG [6,8,35]. In addition, muscle activity, such as microvibrations [9] and muscle tension [10,36], is affected by stress. The Sympathetic Nervous System (SNS) of the Autonomous Nervous System unconsciously controls these vital signs and are thus considered reliable sources [37]. Prior work demonstrated EDA as being linearly related to arousal and widely used in the context of stress sensing [38,39,40]. More specifically, EDA has been used to measure stress in applications, such as measuring the stress of call centre agents [39] and the discrimination of stress from the cognitive load [40]. In addition, in many laboratory and field studies, EDA is considered one of the gold-standard methods to sense stress [41]. As the SNS mainly controls EDA, it is regarded as a reliable physiological sign for acute stress [42].

Furthermore, prior work revealed that mental stress related to cognitive load has an impact on HRV [6,8], in particular towards reduced HF components [43,44,45]. Measurable changes in HR has also been observed during high attention tasks [44]. Electrocardiography (ECG) and Photoplethysmogram (PPG) [46,47] are the earliest technologies used in literature to measure HR and HRV. High power consumption and tight sensor placements are the major draw back of these technologies. Meanwhile, various studies, such as BioWatch [48], SenseGlass [49], SeismoTracker [50], investigated smart devices capable of measuring HR and HRV based on ballistorcardiography (BCG). Although BCG can compete with state-of-the-art techniques [51], it is susceptible to unwanted motion artifacts and thus only reliable in resting states, such as sleeping, standing or sitting. In summary, wearable sensing using wristbands, nail clips, vests and headbands are often uncomfortable, given the bulkiness and tight sensor mounting.

Another option is contact-free sensing of physiological data [52], such as using thermal cameras [53,54] or Doppler radar [55]. As cameras are typically expensive, webcams are a low-cost alternative to measuring HR and HRV from the human face [17,18]. Inspired by previous studies, COGCAM [16] measured cognitive stress with digital cameras, placed 3m away from the user. In these studies, participants are instructed not to move their head, which restricts natural behaviours. An ambient light source ensuring constant light conditions is essential. Privacy concerns may arise when using cameras for tracking.

An increased muscle activity can also indicate stress. For instance, an increased amplitude of the muscles’ microvibrations [9] and an increased muscle tension can provide stress indicators [10]. Other researchers [41,56,57] have detected some types of muscle tension implicitly by analysing keyboard and mouse control. An increased typing pressure and a greater contact with the surface of the mouse has been observed in stressed conditions [41]. We believe such implicit sensing is an unobtrusive and thus desirable approach we would also like to explore.

#### 1.1.2. Facial Expressions

In recent years, identifying affective states based on facial expressions has been widely explored in the area of affective computing [58]. Technology-wise, vision-based camera tracking, including depth cameras [59], are the most commonly used technologies in identifying facial expressions [60]. A recent work [61] investigated stress and anxiety sensing using facial cues, in which the authors’ induced acute stress by internal and external stressors. All videos recorded were analysed posterior and a machine learning model scored between 80–90% accuracy in recognizing stress tasks. Although vision-based identification is demonstrably effective, unfavourable light conditions and movement artifacts easily affect detection accuracy.

Other researchers identify facial expressions based on skin-contact electrodes, such as piezoelectric sensing [62], EMG [63,64], capacitive sensing [65,66] and electric field sensing [67]. These researches demonstrate the detection of facial gestures, such as frowning, eye wink, eye-down, mouth movements, etc. and infer on emotions such as frustration, confusion and interest engagement. Although the identification of emotions and facial expressions seem reliable, the outcome may differ in real-life scenarios given that facial expressions are known to deceive easily [68].

## 2. StressFoot

In this paper, we explore the feasibility of utilising foot movements and posture characteristics to identify acute stress.

### 2.1. Concept

Previous studies demonstrate that body expressions are as powerful as facial expressions in conveying emotions [13,69,70]. Body expressions can play an important role in non-verbal communication than previously thought [69,71]. Studies have also utilized body expressions to detect stress [72]. According to Wallbott et al. [73], changes in body posture provides a strong indication for the changes in affective states. Body expressions are stated as being greater in revealing a deception than relying on facial expressions [68]. Some affective expressions may even be better pronounced using body posture than facial expressions [74]. Recent advances in ubiquitous computing enable the recognition of these body expressions. For instance, computer games such as Nintendo Wii and Microsoft Kinect [13,75] not only utilise body movements as a means to control the game but also to capture the emotional and cognitive performance of the player. The majority of previous works on affective recognition systems utilising body postures and body expressions rely on vision-based techniques that analyse motion data by rgb and depth cameras [75,76]. For instance, Kleinsmith et al. [75] classified four affective states (concentration, defeat, frustration and triumph) with people playing a sports game on Nintendo Wii, and achieved an accuracy of 50%. Another work demonstrates the detection of sadness, joy, anger and fear with a 84–94% accuracy using a 6-camera Vicon motion tracking system. Although vision-based systems seem promising in identifying body postures, these are impractical to integrate into one’s daily routine when aiming to identify emotions, such as those induced by acute stress. As an alternative for vision-based approaches, inferring stress based on posture has been explored previously by embedding pressure sensor in chairs [77]. In this work, authors identified fast movements of Centre of Pressure as a valuable feature in detecting stress. A similar approach was used to detect interest levels of students [78]. This approach required instrumenting chairs with high-resolution pressure sensors, which may not be scalable. However, we believe the sitting posture variations should reflect from the foot posture and motion variations. In literature, smart insoles has been used to detect postures unobtrusively [23,79]. Hence, a smart insole-based solution could be a practical and unobtrusive solution to identify stress that is induced by body language, such as through tracking leg movements and posture characteristics.

### 2.2. Prototype

**Figure 1:**
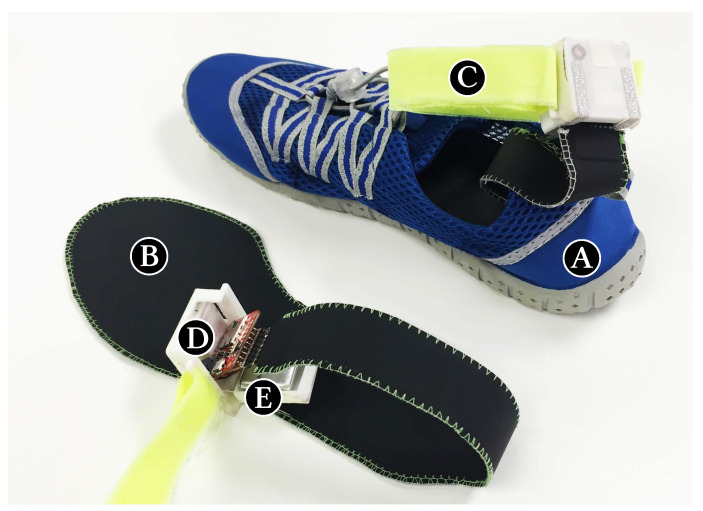
Prototype: (**A**) shoe, (**B**) sensing.tex insole, (**C**) ankle strip, (**D**) SAMD21 microprocessor board with inertial measurement unit (IMU), secure digital (SD) card, voltage divider circuit and (**E**) 400 mAh Battery.

The base of the StressFoot prototype is a pair of sport shoes (Figure 1A). To sense the pressure distribution of the foot, we insert a pair of pressure sensitive insoles (UK size 9–10). These insoles are commercially available from sensing.tex [80] and offer 16 pressure sensors that rely on a force sensitive resistance technology (Figure 1B). In addition, the sensor placement is aligned with the critical pressure points discovered in previous work [81,82]. To drive the insoles, we developed a voltage divider circuit interfacing the insole with the Sparkfun Razor board that consists of an SAMD21 microprocessor manufactured by Atmel Corporation in San Jose, CA, USA. To greater understand the angle and motion of the leg and feet, we also queried the accelerometer of 9 DOF IMU (MPU-9250) manufactured by InvenSense in San Jose, CA, USA that comes with the microcontroller board, which was tied to the user’s ankle. We decided to only utilise the Accelerometer (X,Y,Z) data, since it provides sufficient information. The prototype has a 3.7 V, 400 mAh LiPo battery and an SD card, to allow a fully wireless operation, without constraining the user’s movements. The prototype can be used for approximately 16 h when it is fully charged.

### 2.3. Features

We ran a pilot study with 5 participants, in which we asked the users to complete the minesweeper game in a limited time. We presented a clock to the user, and an observer was also present to make negative comments on the user’s performance. Among all participants in the stress situation, we observed a change in leg posture and a different foot contact with the floor. Two participants demonstrated a nervous leg shaking and foot tapping. Our prototype is designed to identify these unique characteristics by sensing plantar pressure distributions of the foot, as well as tracking the foot angle and motions. We defined 10 low-level features reflecting these behaviours, which are:

#### 2.3.1. A: Foot Pressure

**Figure 2:**
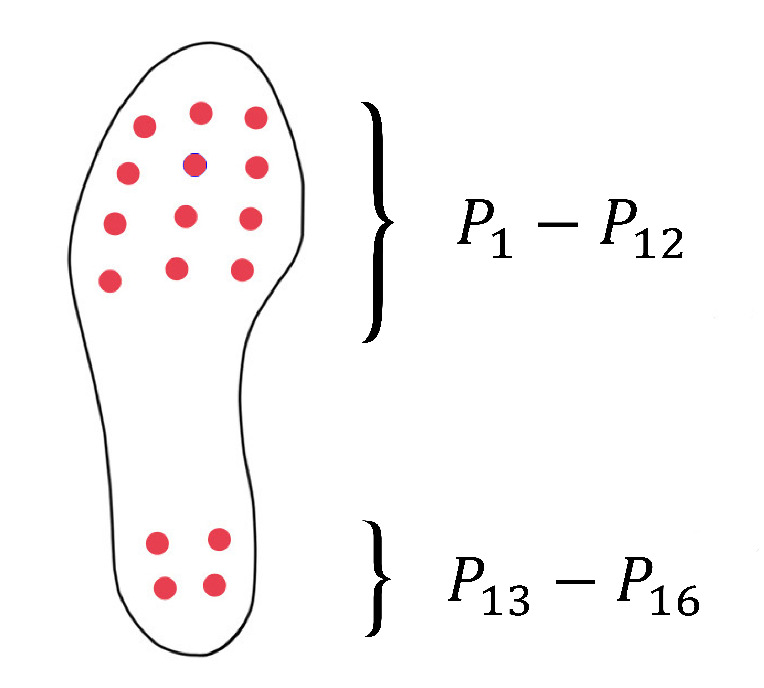
The sensor layout of the insole.

The forefoot pressure was calculated by adding the pressure of 12 sensor points located at the forefoot area of the insole. To calculate rear-foot pressure, the sensor readings of 4 sensors located at the heel area were added together. The sensor layout is depicted in Figure 2.

If the *i*th sensor is $P_{i}$:

(1) $$P_{F}=\sum_{i=1}^{12}P_{i}$$

(2) $$P_{R}=\sum_{i=13}^{16}P_{i}$$

Before summing the raw data, we applied a moving average filter of a 1 s window size to filter out high frequency noises. Finally, we calculated the total foot pressure by summing up all pressure points.

(3) $$P_{T}=\sum_{i=1}^{16}P_{i}$$

Since the features are independent from previous data points, all pressure features were segmented into 10 s non-overlapping windows. Then, the mean of each window is used for the model training.

#### 2.3.2. B: Centre of Pressure

**Figure 3:**
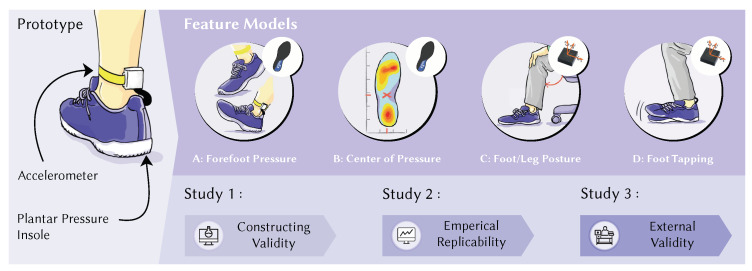
To detect acute stress, we present a shoe prototype incorporating a pressure sensitive insole and an IMU worn around the ankle. Based on literature research and observations, we propose four features that can reveal the user’s stress level. We show that a significant forefoot pressure, deviations in the centre of pressure, different foot/leg postures, and tapping of the foot, can indicate an elevated stress level. To validate generalisability, we conducted three studies: Study 1—Constructing Validity, Study 2—evidencing Empirical Replicability and Study 3—testing for External Validity. Our proposed models score reasonable accuracy and show robustness across different users.

Literature has shown the Centre of Pressure (CoP) as an interesting feature to understand different postures and motion while standing [23], as well as sitting [77]. Therefore, we calculated the CoP for both dimensions (X-axis and Y-axis—see also Figure 3B) by using a weighted average calculation as mentioned in prior work [23]. The CoP was also calculated over 10 s of non-overlapping windows since it is independent from the previous window.

(4) $$Y_{CoP}=\frac{\sum_{i=1}^{12}P_{i}y_{i}+3\sum_{i=13}^{16}P_{i}y_{i}}{P_{F}+3P_{R}}$$

(5) $$X_{CoP}=\frac{\sum_{i=1}^{12}P_{i}x_{i}+3\sum_{i=13}^{16}P_{i}x_{i}}{P_{F}+3P_{R}}$$

#### 2.3.3. C: Foot/Leg Posture

The acceleration data of each axis (aX, aY, aZ) primarily indicates the leg/foot posture defined by the knee angle as seen in Figure 3C. Similar to the above mentioned features, the raw data stream of each axis was segmented into non-overlapping 10 s windows and the mean of the each window was used in model training. Since the sampling rate was 50 Hz, the mean of 500 data points were taken in each 10 s window for all 3 axes. If the number of data points per window is *W* where W=500:

(6) $$aX_{mean}=\frac{1}{W}\sum_{i=1}^{500}aX$$

(7) $$aY_{mean}=\frac{1}{W}\sum_{i=1}^{500}aY$$

(8) $$aZ_{mean}=\frac{1}{W}\sum_{i=1}^{500}aZ$$

#### 2.3.4. D: Foot Tapping

The dominant and the median frequency of each axis are used to describe foot motions, such as foot tapping, waving and leg shaking. We recognised that some motions may be very minimal. To avoid cancelling out these low-amplitude motions when calculating a 3D vector-norm, we deployed the axis-inversion algorithm Matthies et al. introduced [83]. For instance, if we inverted X axis of the accelerometer the inverted axis $aX_{inv}$ would be: $aX_{inv}=2MAX\left(aX\right)-aX$. After segmenting the signal into 10 s non-overlapping windows, we calculated three 3D norms, each with a different inverted axis. From the resulting signals, the 3D-norm with the highest peak-to-peak, $V_{3D}$ was selected for further processing. Then, FFT was used to identify the frequency components of the signal before we extract the dominant and median frequency [84].

(9) $$F_{D}=F_{MaxEnergy}\left(FFT\left(V_{3D}\right)\right)$$

(10) $$F_{M}=F_{MedianEnergy}\left(FFT\left(V_{3D}\right)\right)$$

## 3. Construct Validity—Study 1

As elaborated before, conceptual-wise leg/foot postures and movements should yield information that could indicate stress. Therefore, in our first study, we aim to construct the validity of this concept by developing a machine learning model that aims to distinguish acute stress and relaxation. The study protocol was approved by the University of Auckland Human Participants Ethics Committee.

### 3.1. Participants

The participants had to be at least 18 years of age, English speaking and able to provide written consent. In addition, participants needed to have a foot size of UK 9–10 to match the size of the prototype. Participants with prolonged foot-related injuries were excluded. We recruited 23 healthy participants (12 Males and 11 Females) aged between 21–32 years old (*M* = 26.4, *SD* = 3.17).

### 3.2. Apparatus

**Figure 4:**
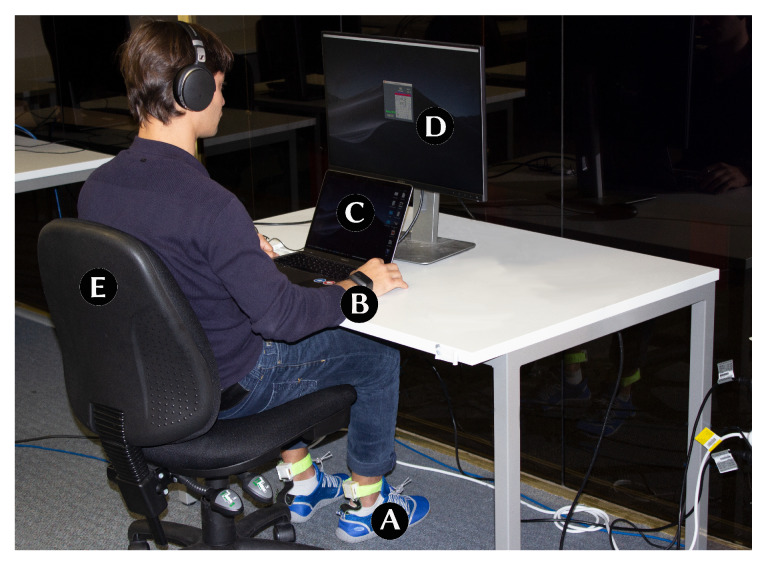
Experimental setup: (**A**) StressFoot prototype, (**B**) Empatica E4 wristband, (**C**) Macbook Pro, (**D**) Dell LED monitor and (**E**) adjustable chair.

To collect the data, we used the StressFoot prototypes. In addition, the Empatica E4 wristband [85] was used to collect the participant’s EDA. All the study tasks were executed and visualised on a 13″ Macbook Pro, which was connected to an extended DELL LED monitor. (see Figure 4).

### 3.3. Procedure & Tasks

After explaining the aim of the study, participants were required to fill out consent forms. Participants were informed that the study aims to explore behavioural changes while performing tasks which induce stress and relaxation. We then collected demographic data and asked the participant to wear the apparatuses. The participants were then asked to sit in-front of the Macbook Pro with an extended display as shown in Figure 4. We allowed the user to adjust the chair and the distance of the table according to their personal preference. During the study, they were also allowed to cross their legs. However, we asked them not to take the foot up to the chair. Once the setup was complete and the user was comfortable, we asked them to complete four tasks. The first, as well as the third task, were stress-inducing tasks, while the second and fourth tasks intended to create relaxation. The task order remained consistent for all participants. After the completion of each task, the participant had to fill a questionnaire. On a 7-point Likert scale, the users had to rate their perceived stress level, energy level and how pleasant they found the task. A NASA Task Load Index (NASA TLX) [86] was conducted to calculate the overall workload per task.

#### 3.3.1. Task 1 [Stress]: Stroop Color and Word Test

The Stroop color and word test is a common stress test (Stroop Test) [87] used in many previous works [10,88,89]. For our study, we customised an open source MatLab-based Stroop Test tool to display four words (“Red”, “Magenta”, “Green” and “Blue”) on the screen. Participants were then asked to type the first letter of the colour (‘R’, ‘M’, ‘G’, ‘B’) the displayed word was coloured with. After each answer, the program provides the result by displaying the word “correct” or “incorrect”. The participant has to perform 5 rounds. Each round has 20 words with a mismatch of 80% between the word and the colour it is printed with. We use several tactics to induce stress among participants. As participants were required to provide answers as quickly as possible, we first induced stress by providing performance feedback on their speed and accuracy. We further elevated stress levels by exposing the participant to loud traffic noises through a headset. Moreover, an experimenter continuously observed the participant, and verbally commented on the participant’s performance. Completing the stress task took 5 min.

#### 3.3.2. Task 2 [Relaxation]: Minesweeper Introduction Video

The second task aimed to induce relaxation among the user after the stress task. It was also intended to provide an introduction to the game, Minesweeper. For this, we selected an entertaining Minesweeper Let’s Play Video from the platform youtube.com. The selection of this video was completed beforehand with 3 pilot study participants. We explicitly informed the user that the video is purely for relaxation purposes and also encouraged the user to relax and enjoy the video. The video took 5 min.

#### 3.3.3. Task 3 [Stress]: Minesweeper

Previous studies already utilized Minesweeper to induce a stress condition [90,91]. Therefore, we used an implementation, in which the user had to find 10 mines in an area of 10 × 10 fields within a time frame of 100 s. The user had to complete 10 rounds. To increase the stress level, the given time was reduced by 10 s every round the user successfully completed the task. The score was indicated at all times, and the user was challenged to complete at least 7 rounds successfully, which was deemed to increase stress. Moreover, similar to task 1, the experimenter provided verbal comments aiming to further elevate the stress level.

#### 3.3.4. Task 4 [Relaxation]: Nature Video

This is the second relaxation task, in which we presented a relaxing nature video for 5 min. Similar to the previous relaxation task, we selected this video based on user feedback from a previous pilot study.

### 3.4. Data Gathering

The participant’s plantar pressure data, as well as the accelerometer data, were collected as a time series with a sampling rate of 50 Hz and stored with the timestamp on an SD card. The EDA data was collected with a sampling rate of 4 Hz at the Empatica E4 wristband. From this data, the mean EDA and the average slope over a non-overlapping windows of 10 s were calculated. In addition to collecting sensor data, the questionnaire collected the user’s perceived stress level, energy level and the level of pleasantness: “How much did you feel stressed during the task? How energetic did you feel during the task? How pleasant did you find the task?” We quantified the answers by letting participants rate their answer on a 7-pnt Likert scale (1: low, 7: high). Moreover, we conducted the NASA TLX to account for the induced task load. It consists of 6 questions which are equally weighted.

### 3.5. Data Analysis

To identify whether our task induced stress or relaxation as we expected, we conducted a pairwise *t*-Test on user’s perceived stress level, energy level, the level of pleasantness and the task load. In addition, to detect significant difference of average EDA slope we conducted another pairwise *t*-Test. Since the data was normally distributed according to Shapiro Wilk’s test, parametric tests such as pairwise *t*-Test and oneway-Anova were used. The participant’s plantar pressure data and IMU data were used to calculate features as mentioned above and used in model training.

### 3.6. Results

**Figure 5:**
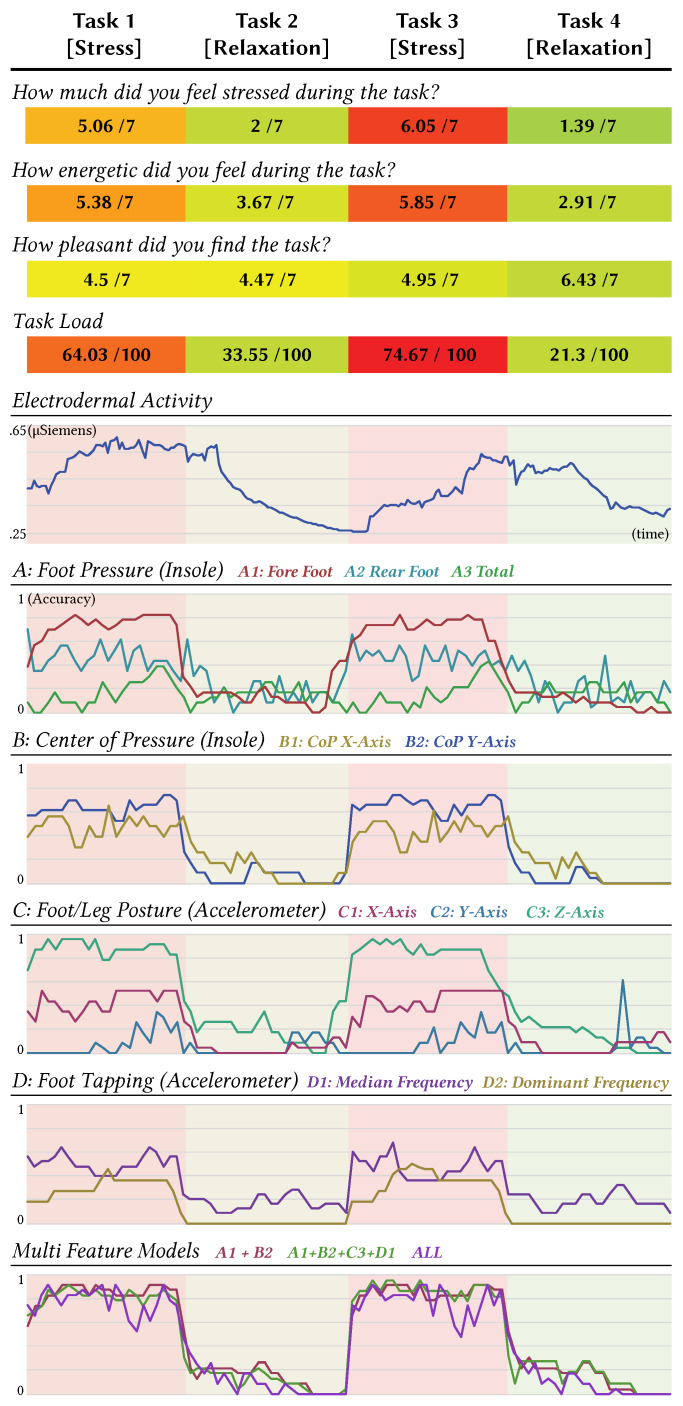
Summary of the results for each task in study 1. The graph of electrodermal activity was computed using the average of the mean electrodermal activity (EDA) over non-overlapping windows of 10 s of all the participants. Accuracy graphs were computed by calculating the average stress detection accuracy of all the participants over non-overlapping windows of 10 s.

As shown in Figure 5, both stress tests, Task 1: Stroop Colour and Word Test, as well as Task 3: Minesweeper, indeed induced an elevated stress level based on the users’ subjective rating. Task 1 scored *M* = 5.06 (*SD* = 0.68) and Task 3 scored *M* = 6.05 (*SD* = 0.76), respectively. In both relaxation tasks, Task 2: Minesweeper Introduction Video, as well as Task 4: Nature Video, the average rated stress level significantly reduced to *M* = 2 (*SD* = 0.76) and *M* = 1.39 (*SD* = 0.58). A pairwise *t*-Test (*t* = 19.33; *p* < 0.05), demonstrating significance and provides proven evidence of our success in inducing stress and relaxation among the users.

Acute stress raises adrenaline levels, making participants feel energetic. In accordance with the answers, all participants stated they felt significantly more energetic during the stress task 1 (*M* = 5.38; *SD* = 0.89) and 3 (*M* = 5.85; *SD* = 1.04), than at the relaxation task 2 (*M* = 3.67; *SD* = 1.67) and 4 (*M* = 2.91; *SD* = 1.78). A pairwise comparison between the stress and relaxation task demonstrated a statistical difference (*t* = 7.09; *p* < 0.05). We hypothesised that a relaxation task will also be more pleasant than a stress task. The perceived pleasure was positive throughout all tasks (T1: *M* = 4.5; *SD* = 1.51, T2: *M* = 4.47; *SD* = 1.6, T3: *M* = 4.95; *SD* = 1.9, T4: *M* = 6.43; *SD* = 0.79). Grouping both relaxation tasks together demonstrated significance to the stress tasks (*t* = 2.84; *p* < 0.05). However, this effect is only significant because the last relaxation task, watching a nature video, was rated as extraordinarily pleasurable. A oneway-ANOVA (*F*_*3,71*_ = 7.88, *p* < 0.05) confirmed this task as significantly more pleasurable than all other tasks. In addition, The NASA TLX seems to be correlated with the actual stress level. A pairwise *t*-Test (*t* = 13.24; *p* < 0.05) indicated two groups as significantly different from each other. Both stress tasks (T1: *M* = 64.03; *SD* = 17.46 and T3: *M* = 74.67; *SD* = 11.32) showed a significantly increased task load in comparison to the relaxation tasks (T2: *M* = 33.55; *SD* = 11.78 and T4 *M* = 21.3; *SD* = 8.08). The data coincides with the self-perceived stress level.

#### 3.6.1. Electrodermal Activity (EDA)

As displayed in Figure 5, the average EDA profile of the participants show a significant increase in both stress tasks (T1 and T3) in comparison to the relaxation task (T2 and T4), which is evidenced by a pairwise *t*-Test (*t* = 2.05; *p* < 0.05). These findings show that the physiological response also correlates to the self-reported stress level and with the Task Load of the participants.

#### 3.6.2. Model Training

As previously stated, we identified four general characteristics that occurs at our foot when placed under pressure. Since our data is low-dimensional to identify the quality of these features, we developed a model for each individual feature using a machine learning classifier. We tested 5 different classifiers with our data, which were: Random Forest (RF) (ntree = 3, mtry = 2), K-Nearest-Neighbour (KNN) (k = 9), Support Vector Machines (SVM) (sigma = 13.9779 C = 0.25, kernel = radial), Decision Trees (CART), cp = 0.02294894 and Linear Discriminant Analysis (LDA). A one-way ANOVA for correlated samples (*F*_*4,60*_ = 4.82, *p* < 0.05) showed significant differences. A Tukey’s post-hoc analysis suggested that LDA-KNN, LDA-SVM, LDA-RF pairs yielded a significant difference. We selected the *LDA* classifier because it showed a consistent and higher mean performance with our data.

Using a supervised learning approach, we trained 10 single-feature models (A1, A2, ..., D2) based on the annotation of our ground truth data. For the ground truth data, we considered the data gathered from all participants, whose stress rating was *M* > 4 for the stress tasks and whose stress rating was *M* < 4 for the relaxation tasks. Hence, for training our model, we excluded ambiguous data, which showed a low stress level at stress tasks (T1: 7/23p., T3: 3/23p.) and an elevated stress level at a relaxation task (T2: 8/23p., T4 0/23p.). Moreover, we excluded the first 60 s and the last 60 s of each task when training our model. Exclusion was necessary to reduce possible noise created by a task accustomisation at the beginning and task exhaustinction at the end of the task.

#### 3.6.3. Model Validation

Each model (*A: Foot Pressure, A1: Fore Foot, A2: Rear Foot, A3: Total Foot, B: Center of Pressure B1: CoP X-Axis, B2: CoP Y-Axis, C: Accelerometer C1: X-Axis, C2: Y-Axis, C3: Z-Axis, D: Foot Tapping, D1: Median Frequency, D2: Dominant Frequency*) was trained and validated using a leave-one*_User_*-out method. Meaning, we built a user-specific model, which was trained by all other users, but does not include the one we tested the model with. Figure 5 depicts the accuracy rates for a stress detection. Instead of calculating an overall accuracy for each task, we calculated the accuracy for non-overlapping windows of 10 s to allows us to observe the progressing confidence throughout the task. Table 1 summarises the overall accuracy rates across all single feature and multi-feature Models. Creating a model using all features provides a reasonable accuracy (*M* = 83.9%). However, the standard deviation (*SD* = 12.01) is higher than any other model. The highest accuracy (*M* = 85.32; *SD* = 8.1) was from the combination of all four high performers (A1+B2+C3+D1). Although the standard deviation is relatively high as a single feature model, C3 showed the highest accuracy (*M* = 83.1; *SD* = 11.9) compared to other single feature models. In addition, a pair-wise *t*-Test was conducted to identify the separation sharpness between the stress and relaxation tasks. Except for model A3, all other models showed a high distinguishability (See Table 1).

## 4. Empirical Replicability—Study 2

To validate the generalisability of our models, we replicated the previous study with different parameters, such as using different users and different tasks. The study protocol was approved by the University of Auckland Human Participants Ethics Committee.

### 4.1. Study Design

The apparatus and data gathering remained the same. The procedure was very similar, with the only difference being the deployment of a single stress and relaxation task. After each task, the participants were asked to answer the same questionnaire from the previous study and to complete a NASA TLX. The data analysis remained the same. We recruited 11 new participants (7 males and 4 females), aged between 22–34 (*M* = 26.4, *SD* = 3.17) with different ethnicity. The inclusion/exclusion criteria were similar to previous study. We also utilised a different stress (Task 5: Mental Arithmetic Test) and relaxation (Task 6: Nature Video) task.

#### 4.1.1. Task 5 [Stress]: Mental Arithmetic Test

Participants were asked to complete 20 challenging maths questions based on fundamental mathematics within 5 min. We created additional pressure by informing participant’s their performance will be graded. Similar to previous tasks, an experimenter observed their performance and commented on their performance.

#### 4.1.2. Task 6 [Relaxation]: Nature Video

To relax the user, we showed a 5 min nature video with soothing music. The video was different to the one in Task 4.

### 4.2. Results

**Figure 6:**
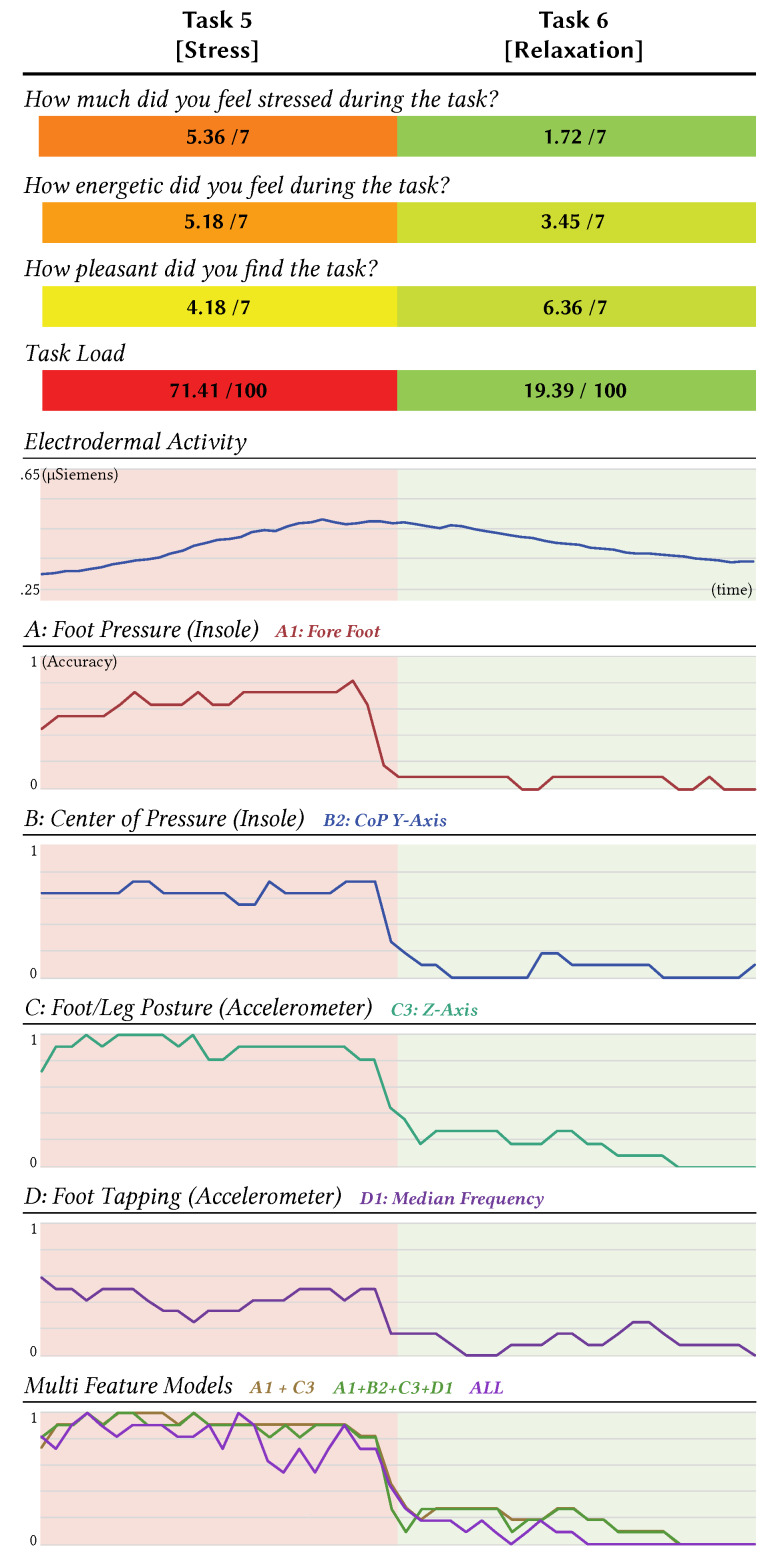
Results of generalisability. The graph of electrodermal activity and accuracy shows the averages of the 11 participants over non-overlapping windows of 10 s.

In accordance with the questionnaire, all the participants agreed they felt stressed (*M* = 5.36; *SD* = 0.92) during the mental arithmetic task and relaxed (*M* = 1.72; *SD* = 0.64) during the relaxation task (see Figure 6). A pairwise *t*-Test (*t* = 11.74; *p* < 0.05) confirmed that all participants were significantly more stressed during stress Task 5 compared to the relaxation Task 6. All participants stated they felt more energetic during stress task 5 (*M* = 5.18; *SD* = 1.47), than at the relaxation task 6 (*M* = 3.45; *SD* = 2.01), which was confirmed as statistically different following a pairwise *t*-Test (*t* = 2.55; *p* < 0.05). Additionally, participants rated the relaxed task (*M* = 6.36; *SD* = 0.67) as more pleasant than the stress task (*M* = 4.18; *SD* = 2.13), which was evidenced as significant by a pairwise *t*-Test (*t* = 3.54; *p* < 0.05). Most crucially, in terms of Task Load (equally weighted), a pairwise comparison (*t* = 9.49; *p* < 0.05) between both tasks clearly indicated that stress Task 5 significantly induced a higher task load than relaxation Task 6.

#### 4.2.1. Electrodermal Activity (EDA)

These subjective ratings are also consistent with the physiological data gathered—the average EDA response. It showed an overall positively increasing slope at the stress task 5 and negatively decreasing slope for relaxation task 6. Comparing the trends of both tasks by a *t*-Test confirmed the average EDA slopes to be significantly different (*t* = 6.9; *p* < 0.05).

#### 4.2.2. Overall Model Validation

The features were extracted as mentioned before and classified (using *LDA*) with seven previously built models. We chose four single feature models based on the best performer for each observed characteristic, which are: A1, B2, C3 and D1. Furthermore, we used a multi-feature model combining all four. The sixth model was generated based on previous data and by combining the most meaningful features of A1 and C3. The final model was a combination of all computed features. Again, we windowed the accuracy of models over 10 s to observe any periodic trends (see Figure 6). The summary of the overall accuracy rates are shown in the Table 2. A one-way ANOVA for correlated samples (*F*_*6,339*_ = 12.75, *p* < 0.05) showed significant differences between the accuracy of the models. A Tukey’s post-hoc analysis revealed that the significant difference occurred due to the low mean accuracy of model D1. Since model A1+B2+C3+D1 showed slightly higher accuracy than model C3+A1 and a lower standard deviation (*M* = 87.45; *SD* = 8.5), we suggest it is the best performing model. Furthermore, it will work for most of the users, since it considers a feature related to foot tapping such as D1. The model incorporating all features showed an overall accuracy of 85.6%, but with a comparably high standard deviation (*SD* = 12.0). Model C3 showed the highest accuracy as an individual feature model (*M* = 86.7; *SD* = 10.0). In a pair wise *t*-Test, all models indicated high separation sharpness between stress and relaxation (*p* < 0.0001).

## 5. External Validity—Study 3

In this study, the goal is to demonstrate the robustness of our model. Therefore, we conducted an in-field study at which we recorded data over a working day from users performing their usual everyday office tasks. Similar to the previous studies, the University of Auckland Human Participants Ethics Committee approved the protocol.

### 5.1. Participants

We recruited 10 participants (7 males and 3 females) aged between 25 and 34 (*M* = 29.9, *SD* = 3.4). Among these participants, four were from the first study, another four were from the second study and two were newly recruited. A requirement for our participant selection was a foot size that matched the prototype. In addition, the participant had to work in an office and spent the majority of the time in a sitting posture (>70%). Apart from this, the inclusion/exclusion criteria remained similar to previous studies.

### 5.2. Task and Procedure

The study was conducted on a particular day that the participants expected to have some periods of acute stress. Some of the stress tasks included working for a deadline, debugging a firmware/software, having a meeting with their supervisor, writing a paper for an upcoming submission, etc. Having lunch/coffee with friends and having casual chats with friends were some activities that would supposedly relax the participants.

The study began at 09:45 a.m. local time at the participants’ office space. After elaborating on the study procedures, filling consent forms and collecting demographic data, the experimenter asked participants to wear the StressFoot and E4 wristband. At 10:00 a.m., the experimenter initialised apparatuses for data collection and asked participants to fill a questionnaire, asking them to rate their current stress level, energy level and how pleasant they felt on a 7-point Likert scale. Then, participants were asked to continue their work as usual. We asked participants to fill out two forms, a calendar application reporting the type and duration of tasks performed, as well as a questionnaire. These were the only tasks we asked the participants to perform hourly over 8 h from 10:00 a.m. to 6:00 p.m. To ensure the participants would remember to report and to fill out the calendar and the form, they received reminders via visual and audio pop-ups at the end of each hour. The questionnaire asked the participants to rate their perceived stress level, energy level, as well as how pleasant they felt during last hour. Part of the questionnaire was to fill a NASA TLX, which we used to calculate the overall workload.

### 5.3. Apparatus and Data Gathering

Similar to the previous studies, the StressFoot prototype was used to collect foot motion and pressure data. The Empatica E4 wristband was used to collect EDA, as well as motion data from the participants.

The data gathering was thus similar to the previous study. Additionally, we collected accelerometer data from the E4 wristband to identify motion (walking) and posture (sitting/standing). We used the E4’s preset sampling rate of 32 Hz. The data analysis also remained similar to the previous study. Next, we segmented the accelerometer data into non-overlapping windows. Literature suggests using a window size less than 10 s, such as 2.5 s [92] or even 1 s [93]. Through an experimentation with three users, we determined 5 s as a suitable window size for our use case. To identify motion and posture, we relied on a threshold analysis with a single feature, as suggested by Gjoreski et al. [94]. The feature used is the first derivative over the entire window, also known as an “Acceleration Vector Change” (AVC) [94]. This feature can identify: walking (AVC >= 2 ms^−3^), standing (AVC < 0.1 ms^−3^) and sitting (0.1 ms^−3^ =< AVC < 0.2 ms^−3^). We applied this to extract the timestamps to identify the sitting time. Using these timestamps, we selected the sitting data from the corresponding foot motion and foot pressure data from the StressFoot prototype.

Finally, the sitting data was segmented into 10 s of non-overlapping windows. Then, all features were extracted, as mentioned before, and classified using the best performing model, which was a multi-feature model (A1+B2+C3+D1). At every 10 s window, the model classifies whether a participant is stressed or relaxed. We then calculated the percentage of the number of windows that classified stressed. This provides a measurement of the duration that a participant might have been stressed. Therefore, for each hour, we calculated a ratio (*R_S_*), where:

(11) $$R_{S}=\frac{nWindows_{\mathrm{sitting}}\left(Stressed\right)}{nWindows_{\mathrm{sitting}}\left(Total\right)}$$

### 5.4. Results

#### 5.4.1. Activities

As depicted in the Table 3, all the participants spent the majority of their time in a sitting posture (*M* = 79.5, *SD* = 6.1). Designing PCBs, coding, soldering, debugging firmware/software, reading/writing papers, meetings, writing emails, having coffee/lunch and watching YouTube, were the major activities that participants reported doing during the study.

Most of the participants marked higher stress levels (>4 on a 7-pnt Likert scale) when they performed office work-related activities, such as coding, debugging and writing papers. A summary of the self-reported stress level, level of energy and level of pleasantness across all participants is depicted in Table 4. According to the table, when participants felt stressed (Stress Level > 4), a lower level of pleasantness (*M* = 3.8, *SD* = 1.4) was reported compared to when participants were relaxed (Stress Level < 4). Welch’s *t*-test further confirmed that there is a significant difference (*t* = −4.10; *p* < 0.05). Moreover, Welch’s *t*-test showed a significantly higher task load when participants were stressed, compared to being relaxed (*t* = 7.02; *p* < 0.05). These findings confirm that we have collected two significantly different levels of stress (stress and relaxation).

Moreover, the rated “level of energy” was not statistically different when dividing the gathered data into two groups (stressed and relaxed) following the Welch’s *t*-test (*t* = 1.64; *p* > 0.05). This means the perceived level of energy does not relate to stress since energy can be both positive and negative.

#### 5.4.2. Electrodermal Activity (EDA)

Table 5 summarises the average EDA slopes of the participants in stressed and relaxed conditions. When a participant marked the previous hour with a stress level > 4, we considered that hour as a high stress duration. We considered ratings below 4 as a relaxed period. When stressed, the majority of participants (5 out of 6) demonstrated an average positive EDA slope. Comparatively, when participants were relaxed, 8 out of 10 participants showed an overall negative EDA slope. However, none of the participants showed a significantly different mean EDA slope according to Welch’s *t*-test (see Table 5). The limited difference is due to high variances of EDA readings caused by external factors, such as motion artefacts, ambient temperature variances and loose contact of electrodes on the skin.

#### 5.4.3. Overall in Field Validation

**Figure 7:**
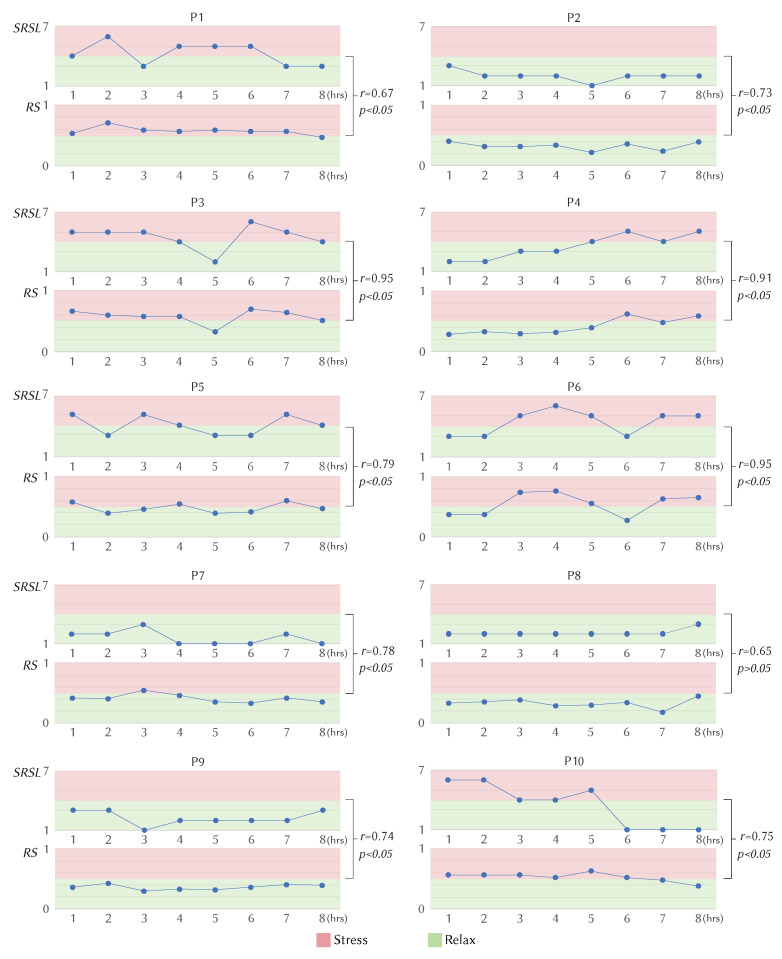
The figure shows the Self-Reported Stress Level (*SRSL*) and *R_S_* for each participant for each hour. Where $R_{S}=nWindows_{\mathrm{sitting}}\left(Stressed\right)/nWindows_{\mathrm{sitting}}\left(Total\right)$. The Pearson’s Correlation Coefficient *r* and the level of significance *p* for each participant’s *SRSL* and *R_S_* is also depicted.

We now analyse how our model compares to the users’ Self-Reported Stress Level (SRSL). Figure 7 depicts the SRSL and *R_S_* of each hour for each participant. The graphs already demonstrate a positive correlation. To identify the significance of this relationship, we calculated the Pearson’s Correlation Coefficients (*r*) for each participant. All participants showed a positive correlation coefficient, with an average of *r* = 0.79 (*SD* = 0.10). Eight out of 10 participants demonstrated a statistically significant positive relationship (*r* > 0.7, *p* < 0.05) between SRSL and *R_S_*. Among these participants, three had a high correlation coefficient (*r* > 0.9) which shows strong relationship between SRSL and *R_S_*. Only two participants showed a moderate Pearson’s Correlation Coefficient (*r* = 0.67 and *r* = 0.65). However, this result was not statistically significant (*p* > 0.05). Even from those two participants, P8 reported lower stress levels (<4) for the entire study duration, which is also confirmed by our model showing a lower *R_S_* (<0.5) value during the entire study period. Overall, we can conclude that all the participants showed a positive correlation, given the majority demonstrated a significantly high correlation between SRSL and *R_S_*. Such findings evidences the robustness of our model in-field, beyond a controlled laboratory setting.

## 6. Discussion

### 6.1. Accuracy

Our highest performing model is an LDA classifier with an accuracy of 86% in laboratory conditions. In literature, the models based on physiological parameters such as EDA, ECG, HRV, showed similar or higher accuracy in laboratory validations [32,39]. For example, an SVM model, based on features from a combination of physiological signals such as EDA, Blood Volume Pulse, ST and PD achieved an accuracy of 90.1% [32]. Although the model has achieved a higher accuracy, it has some limitations in field deployment. PD requires line of cite and may generate privacy concerns. In addition, according to authors, removing PD may drop the accuracy closer to 60%. In another study, authors were able to discriminate stress from cognitive load by using a LDA classifier based on EDA [40]. In a laboratory setting, they achieved an accuracy of 82.8%, which is slightly lower than the results we achieved in our study. In addition, an SVM model based on facial EMG, respiration, EDA and ECG was used to recognise 5 emotional states such as high stress, low stress, disappointment, euphoria and neutral. The paper reports an accuracy of 86% in a laboratory study [95]. In addition, another study reported a system which can classify stress with 86% accuracy based on 15 features extracted from EEG, ECG and EDA signal [96]. All these systems may be inconvenient, given the need to tightly attach multiple wearable sensors onto the body. In our approach, we can recognise stress and relaxation with a similar accuracy by using a simple accelerometer model, such as model C3. However, our current model cannot detect different levels of stress.

Some of the prior studies related to body language [76] study and facial [61] expression also achieved similar or slightly higher accuracy. However, many of these methods used camera-based systems, which may have limitations in real-life implementations. Some of the higher accuracy methods use multiple motion capture cameras, which is impractical to deploy in real-life, specifically in an office environment. For example, a work which detected emotions related to negative stress such as sadness, joy, anger and fear showed an accuracy of 93% [76]. However, the method uses 6 camera vicon motion capture system. Although the accuracy is slightly lower than vision-based methods, our approach captures certain body language related to stress while sitting by using a more practical method, which can be easily used in real-life applications.

On the other hand, there are several real life validations reported in literature. Healey and Picard proposed an LDA classifier to recognise stress of drivers using ECG, EMG, EDA and respiration [4]. Regardless of the cumbersome setup which consists of many on body sensors, they were able to recognise three levels of stress (low, medium and high) in 97% of accuracy. In another study, Hernandez et al. achieved an accuracy of 74% in call-center stress detection [39]. They proposed a person specific SVM model, which uses EDA response for stress detection. In our field study, by using four features related to foot motion and posture, we identified significantly high correlations between self-rated stress levels and model-derived stress levels across users, ultimately showing the robustness of the method.

Overall, methods which utilise a combination of physiological parameters seem to achieve higher accuracy than our proposed method [4,32]. However, sensing multiple physiological parameters requires attaching multiple sensors onto the user, compromising the comfort. Contrarily, previous methods based on single physiological parameter demonstrated either similar accuracy or lower accuracy [39,40]. The lower accuracy could be due to data losses and subjective differences. Some methods based on body language and facial expressions have shown higher accuracy [61,76] due to high sensing accuracy in visual-based sensing. However, those methods seem highly obtrusive in real-life.

In addition, a recent study compared unobtrusive sensors for stress detection at sedentary computer work [97]. In their analysis, they considered wrist worn, chest worn and thermal imaging based sensors. They identified that wrist worn sensors, such as EDA and PPG, may not capture stress accurately due to frequent data losses. This is mainly due to motion artefacts, such as electrode movements, detaching from the skin and a change in pressure on the skin. In addition, chest worn sensors which sense HR showed similar issues due to posture changes generating high noise and thus failing to maintain proper contact with the skin. This is highly problematic in 6–8 h of sensing in a typical working day. However, our approach does not result in such issues, specifically in an office working environment. We have proven that risks of data losses are not present with our method, given we sense foot motion and posture characteristics while sitting. While the study identified that thermal imaging resulted in a greater identification of stress during computer work, this is not always a practical method. Thermal imaging requires a consistent line of sight, which is problematic when the individual needs to attend to tasks away from their typical working desk. Our method poses no such issues.

### 6.2. Applications

#### 6.2.1. Professional Environment

In our studies, the recruited participants mainly performed computer-aided tasks in an office environment while in a sitting posture. Other occupations, such as cashiers, emergency/non emergency call centre workers, also perform their tasks while in sitting posture for prolonged periods. In addition, the working environment of these occupations are similar to an office environment. Hence, our method may work for these occupations as well. In such a scenario, stress sensing could be used to improve mental health. However, further studies need to identify the viability of foot-based stress sensing method for such occupations. In addition, remote stress monitoring of adults under home care is a another potential application. Simple accelerometer can be embedded to a sock and thus monitor both stress and activity level using a single sensor. However, to accomplish this further studies with better classification algorithms stated in literature are required [98,99].

#### 6.2.2. The Quantified Self

Enriching self-tracking with a stress detection is another application. In this community, stress is being triangulated with several data of wearable sensors [100]. A simple feature could substantially increase accuracy. Moreover, using an already available wearable, like a shoe, can address users who prefer not to wear additional garment accessories.

### 6.3. Limitations and Future Work

#### 6.3.1. Quantifying Multiple Stress Levels

Both lab studies only investigated discriminating stress from relaxation without aiming to identify different levels of stress. However, the results of study 3 indicates that the frequency of demonstrating stress related postures could reveal the extent of stress. However, it requires further research to infer on different levels of stress that could be based on the frequency of stress-related foot movements and foot posture characteristics.

#### 6.3.2. Stress Detection in Sitting Posture and Other Activities

In our society, sitting is the most common posture demonstrated during both the working week and weekends [101], which our findings also confirm (see Table 3). Therefore, our proposed models would principally work for a majority of the time, whenever an individual is doing some sort of intellectual work while seated. Detecting stress in other postures exceeds the scope of our current research. Identifying stressful situations when performing regular activities, such as standing, walking and running are thus considered future work. For this, we would first need to identify the current posture and activity, such as by using an insole [24,25] or IMU [92,94,102], as shown in the literature.

#### 6.3.3. Accuracy Boost with Personalised Models

We aimed to develop a generalised model capable of working across different users. We observed that the accuracy of our generalised models decreases for features, such as Median Frequency and Dominant Frequency. This is because not all users demonstrate an elevated foot tapping/shaking when stressed. In addition, we observed that individuals may have slightly different foot posture and motion characteristics depending on the BMI and other personal habits. Relying on a personalised model will further boost accuracy, particularly for features like foot-tapping. Further, a Neural Network approach can be advantageous, particularly when building a personalised model with a high volume of data gathered in the wild.

#### 6.3.4. Improved Hardware

In our current prototype, the IMU (in conjunction with the control unit) was attached to the ankle. Our long-term goal is to integrate all these components into a smart footwear. This may change the orientation of the IMU. Thus, the main signal could spread to different axis, resulting in another axis to provide higher separation sharpness.

## 7. Conclusions

We presented StressFoot, a pair of smart shoes that can sense stress unobtrusively by using a pressure sensitive insole and an IMU. Our prototype is capable of identifying acute stress and relaxation while sitting, such as performing office tasks. We identified four characteristics, which reflect foot pressure distributions, foot posture variations and foot tapping. Based on these features, we trained several machine learning models with 23 participants by using a leave-one_*user*_-out and validated this as a method to detect stress with an average accuracy of ∼85%. Then, with 11 additional participants, we demonstrated the replicability of our model with a similar overall accuracy of ∼87%. Finally, to evidence external validity, we conducted a field study with 10 participants, and evaluated the robustness of our models in an actual office setting. The outcome was that the computed stress level provided by our machine learning model correlates with the self-reported stress level with a coefficient of *r* = 0.79. We envision StressFoot to be an unobtrusive system capable of detecting the user’s stress level on a daily basis. By drawing attention to the user’s mental stress condition, such a system may already be able to contribute to an improvement in overall mental well-being in the future.

## Figures and Tables

**Table 1:** Model performance (selected classifier: Linear Discriminant Analysis (LDA)).

Feature Model	A1+B2+C3+D1	C3+A1	ALL	C3	A1	B2	D1	C1	B1	D2	A2	C2	A3
**Accuracy [%]**	85.32	85.0	83.9	83.1	79.8	78.8	69.3	67.3	66.6	65.3	62.5	52.8	50.7
**SD [%]**	8.1	9.7	12.01	11.9	11.1	6.7	7.9	8.0	11.3	6.7	11.4	10.6	9.25
**Distinguishablity** [***p***]	<0.0001	<0.0001	<0.0001	<0.0001	<0.0001	<0.0001	<0.0001	<0.0001	<0.0001	<0.0001	<0.0001	<0.001	=0.2

**Table 2:** Model Performance for second set of participants for different tasks (Selected classifier: LDA).

Feature Model	A1+B2 +C3+D1	C3+A1	ALL	C3	A1	B2	D1
**Accuracy [%]**	87.45	87.3	85.6	86.7	79.3	79.6	66.5
**SD [%]**	8.5	9.62	12.0	10.0	8.5	6.5	7.6

**Table 3:** Percentage of being in sitting posture of each participant during the 8 h field study.

Participant No:	P1	P2	P3	P4	P5	P6	P7	P8	P9	P10
**Sitting time [%]**	85.7	76.5	76.3	73.9	88.4	86.7	70.6	75.1	83.8	78.2

**Table 4:** Summary of level of pleasantness, energy level and task load.

	Level of Stress > 4	Level of Stress < 4	*p*	*t*
**Level of Pleasantness**	M = 3.8, SD = 1.4	M = 5.2, SD = 0.9	<0.05	4.10
**Level of Energy**	M = 4.9, SD = 1.1	M = 4.3, SD = 1.4	>0.05	1.64
**Task Load**	M = 62.3, SD = 8.6	M = 42.4, SD = 11.4	<0.05	7.02

**Table 5:** Summary of EDA slopes of each participants while being stressed and relaxed.

Parti. No:	Stress	Relax	*p*	*t*
P1	1.99 × 10${}^{-6}$	−1.61 × 10${}^{-5}$	>0.05	0.08
P2	-	−3.58 × 10${}^{-6}$	-	-
P3	5.16 × 10${}^{-6}$	−3.93 × 10${}^{-5}$	>0.05	0.02
P4	2.91 × 10${}^{-6}$	−9.69 × 10${}^{-5}$	>0.05	0.17
P5	7.04 × 10${}^{-5}$	−2.38 × 10${}^{-4}$	>0.05	0.15
P6	2.95 × 10${}^{-5}$	−6.81 × 10${}^{-5}$	>0.05	0.38
P7	-	9.03 × 10${}^{-6}$	-	-
P8	-	−5.77 × 10${}^{-5}$	-	-
P9	-	4.48 × 10${}^{-6}$	-	-
P10	−8.08 × 10${}^{-5}$	−3.52 × 10${}^{-5}$	>0.05	0.14

## Abbreviations

The following abbreviations are used in this manuscript:

IMU	Inertial Measurement Units
CoP	Centre of Pressure
EDA	Electrodermal Activity
SRSL	Self-Reported Stress Level
